# Acquired G2032R Resistance Mutation in ROS1 to Lorlatinib Therapy Detected with Liquid Biopsy

**DOI:** 10.3390/curroncol29090520

**Published:** 2022-09-16

**Authors:** Balázs Jóri, Markus Falk, Iris Hövel, Peggy Weist, Markus Tiemann, Lukas C. Heukamp, Frank Griesinger

**Affiliations:** 1 Lung Cancer Network NOWEL, 26129 Oldenburg, Germany; 2 Institute for Hematopathology Hamburg, Fangdieckstraße 75A, 22547 Hamburg, Germany; 3 Department of Hematology and Oncology, Pius-Hospital Oldenburg, Georgstraße 12, 26121 Oldenburg, Germany; 4 Department of Internal Medicine-Oncology, University of Oldenburg, Georgstraße 12, 26121 Oldenburg, Germany

**Keywords:** NSCLC, lorlatinib, ROS1, resistance mutation, liquid biopsy, G2032R

## Abstract

Lorlatinib, a third-generation anaplastic lymphoma kinase (ALK)/receptor tyrosine kinase inhibitor (ROS1), demonstrated efficacy in ROS1 positive (ROS1+) non-small cell lung cancer (NSCLC), although approval is currently limited to the treatment of ALK+ patients. However, lorlatinib-induced resistance mechanisms, and its efficacy against the resistance mutation G2032R in ROS1, respectively, have not yet been fully understood. Furthermore, concomitant tumor suppressor gene p53 (TP53) mutations occur in driver alteration positive NSCLC, but their prognostic contribution in the context of ROS1 inhibition remains unclear. Here we report a ROS1+ NSCLC patient who developed an on target G2032R resistance mutation during second-line lorlatinib treatment, indicating the lack of activity of lorlatinib against ROS1 G2032R. The resistance mutation was detected in plasma-derived ctDNA, signifying the clinical utility of liquid biopsies.

## 1. Introduction

Activating translocations in the c-ros oncogene 1, receptor tyrosine kinase (ROS1), provides a therapeutic target in 1–2% of all non-small cell lung carcinoma (NSCLC) patients [1,2]. The clinical features of these ROS1+, treatment-naïve stage IV NSCLC patients do not differ significantly from other driver mutation positive cohorts. The most common metastatic site is the central nervous system (CNS) [3]. The clinical course of ROS1+ patients is usually deemed to be favorable. With the multitarget ALK/MET/ROS1-inhibitor crizotinib, which received FDA approval in 2016 and became the standard of care, a median PFS of more than 13 months can be reached. A clinical hallmark of ROS1+ patients is an increased risk of thrombotic diathesis, the reason for which is so far poorly understood.

Crizotinib in most patients with ROS1+ NSCLC leads to a therapeutic response; however, in analogy to other TKI’s, relapse ultimately occurs and on target resistance mutations can be identified in 30–50% of patients, of which the most frequent resistance mutation is the G2032R [4,5]. Preclinical data suggest lorlatinib is active against ROS1 G2032R [6], and demonstrates excellent intercranial activity in patients [7]. Therefore, lorlatinib has become a second-line option following disease progression under crizotinib [8]. However, while the IC50 of lorlatinib against WT ROS1 is 0.7 nmol/L, the IC50 against G2032R is 196.6 nmol/L. This is different to the analogous ALK resistance mutation, where the IC50 is 49.9 nmol/L against G1202R, while 2.3 nmol/L in WT [4,5]. Therefore, it is conceivable that the concentrations of lorlatinib in vivo might not suffice to block ROS1 signaling. Consistent with this preclinical observation, lorlatinib did not show clinical activity against G2032R in a small cohort [9] and the G2032R mutation persisted in the patients treated with lorlatinib. Recently, G2032R was discovered in vitro in entrectinib- and lorlatinib-resistant clones, and a similar mutation, G2032K, in a lorlatinib-treated patient, suggesting G2032X is an acquired resistance mutation [10,11]. To our knowledge, the acquisition of a ROS1 G2032R during treatment with lorlatinib has rarely been reported; nevertheless, it is not unknown: similarly to our case, in a study of 55 patients, G2032R was absent in one patient’s post-crizotinib sample and emerged after the lorlatinib treatment [5].

Mutations in the tumor suppressor gene p53 (TP53) are the most common mutations found in NSCLCs and co-occur in about 30% of NSCLC patients with ALK and ROS1 translocations [12]. The negative impact of co-mutations such as TP53 mutations on PFS and OS has been demonstrated in a small series of ROS1 patients and these data are consistent with ALK+ and EGFR mt+ patients [13,14]. When receiving crizotinib, concomitant TP53 mutations in ALK-rearranged NSCLC patients have been associated with unfavorable survival, a reduced response and shorter progression free survival (PFS) in multiple studies [12,15,16,17,18,19]. Data on crizotinib sensitivity and TP53 co-mutated and ROS1-positive NSCLC are limited. One retrospective multicenter study of 94 lung cancer patients with ROS1 rearrangements, in which 33% carried a concomitant TP53 mutation, correlated with worse PFS (median, both NR, *p* = 0.0417) [20]. In addition, in a German study of lorlatinib-treated ALK- and ROS1-positive NSCLC patients, TP53 mutations were associated with a threefold lower PFS of 3.7 versus 10.8 months [12]. However, the impact of concomitant TP53 mutations in this setting requires evaluation in prospective clinical trials.

At the time of disease progression during targeted treatment, the accurate identification of resistance-related genetic alterations is crucial for patient management. However, repeated tissue biopsies of progressing tumor manifestations are not always feasible, and they might not reflect tumor heterogeneity at different sites. Liquid biopsies, conversely, have been recommended for resistance monitoring and the molecular testing of patients with metastatic NSCLC in cases where tissue samples may be insufficient or not available [21].

Approximately 15% of advanced lung adenocarcinomas develop venous thromboembolic events all along the course of the disease. This risk might increase due to molecular subtypes: thromboembolic events have been described in both ALK and ROS1 translocated patients and thus are one of the most eminent symptoms leading to diagnosis and contributing to mortality [22]. The incidence of venous thromboembolic events all along the course of the disease in advanced-stage lung adenocarcinomas is approximately 15%. It is plausible that the different molecular subtypes might influence the risk of thrombosis [23].

In this case report, we present a lung adenocarcinoma patient with a CD74-ROS1 fusion and an inactivating TP53 co-mutation at diagnosis. We show that the ROS1 resistance mutation G2032R, which was not demonstrated at the start of second-line treatment with lorlatinib, emerged while on lorlatinib treatment. In addition, we highlight the clinical utility of liquid biopsy-based hybrid capture NGS.

## 2. Case Presentation

**Figure 1:**
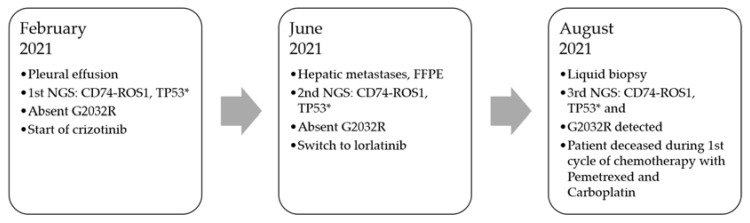
Summary of treatment plan and performed diagnostics.

A 42-year-old, light smoker (9 pack/years), female patient presented in February 2021 with advanced adenocarcinoma of the lung, stage IV (T1cN3M1c (HEP, OSS, PLE, LYM) (Figure 1). Complications were a deep venous thrombosis and a pulmonary embolism. While on therapeutic anticoagulation, the patient developed multiple intracerebral infarcts necessitating treatment with both low molecular heparin and ASS. Due to limited and sparse FFPE tissue, the initial molecular diagnostic was performed using PCR-based methods covering the hotspots in EGFR, KRAS and BRAF and immunohistochemistry analysis (IHC) for PD-L1, ALK and ROS1. The initial analysis resulted a PDL-1 status TPS: 80% and high ROS1-expression in the cytoplasm of tumor cells. The patient started to receive crizotinib treatment on the 24th of February 2021 based on the positive IHC result.

One day later, the hybrid capture-based NGS analysis (HC-NGS) was reported on a pre-therapeutically taken pleural. The analysis confirmed the initial IHC-based diagnosis of ROS1 positivity and revealed a CD74-ROS1 translocation (C7:R34) and an additional nonsense mutation in TP53.

Eight weeks after the start of crizotinib, a CT scan revealed good partial remission of all tumor sites. However, 4 months after the start of crizotinib therapy, the patient progressed, in particular with histologically confirmed hepatic metastases. Because of the liver being the fastest progressing site, a liver biopsy was taken. Molecular analysis of this tumor site in June 2021 by HC-NGS confirmed the initial genetic alterations with no further resistance mutations. In particular, G2032R was not detected. At this time the patient had a fibrinogen as low as 65 mg% and an AT III of 40%; therefore, ASS was paused and heparin was reduced to half dose. The patient was switched to lorlatinib on the 28th of June 2021. Hypofibrinogenemia and low AT III recovered to normal values within 7 days and the patient showed a good response after 4 weeks.

Two weeks later (August 2021), after the initial PR on lorlatinib, the patient progressed again massively in the liver with a liver now reaching in the pelvic region. Due to hypofibrinogenemia, a liver biopsy was deemed to be too risky and a liquid biopsy was performed. Hybrid capture NGS confirmed the previously known ROS1 translocation and TP53 mutation as well as a novel ROS1 p.G2032R resistance mutation. At this time hypofibrinogenemia and low AT III developed again. A first cycle of chemotherapy with pemetrexed and carboplatin was started; however, the patient died of multiple intracerebral thromboses.

## 3. Discussion

### 3.1. Thrombotic Diathesis and Clinical Course

The patient showed thrombotic diathesis and disseminated intravasal coagulopathy with low fibrinogen and AT III at first and second relapse. While the reasons for this hemostaseologic anomaly have not yet been elucidated, fibrinogen and AT III were recovered within 7 days indicating that the hemostaseologic disorder depends on the activity of the tumor and that it is reversible if the tumor responds to therapy.

### 3.2. G2032R Resistance Mutation Development under Lorlatinib

The ROS1 p.G2032R mutation is well known to be selected under treatment with crizotinib, while it has so far only been described in single cases with a ROS1-activating fusion to emerge under lorlatinib’s selective pressure. Additionally, this patient showed massive thrombotic diathesis at diagnosis, which increased during the clinical course of the disease, leading ultimately to death.

**Figure 2:**
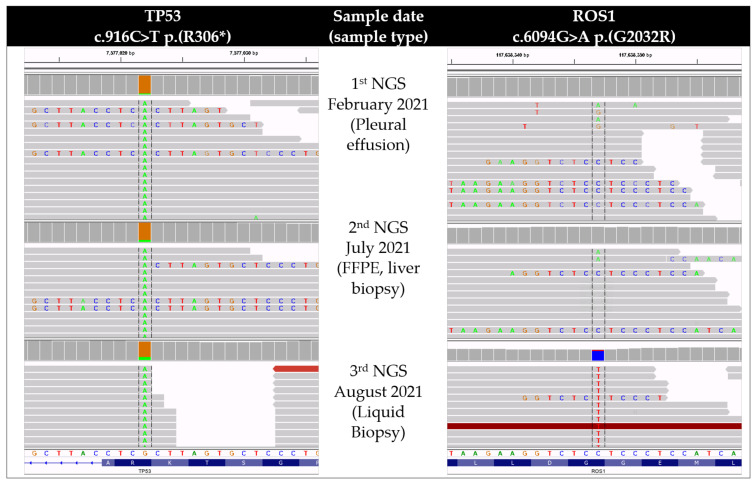
Single nucleotide variants detected with NGS during resistance development. Initial diagnosis detected NM_001126112.2: c.916C > T (p.(R306 *)) mutation in TP53 with a variant allelic frequency (VAF) of 6% at a tumor cell content (TCC) of 70%. It has been confirmed in the second biopsy from the liver 4 months later (TP53 VAF: 12% at TCC: 30%). Neither 1st, nor 2nd NGS showed the presence of a G2032R resistance mutation in ROS1. Five months after the initial diagnosis, liquid biopsy confirmed TP53 mutation (VAF: 19%) and revealed the NM_002944.2: c.6094G > A (p.(G2032R)) resistance mutation (VAF: 11%) in ROS1.

The TP53 mutation (Figure 2) and the CD74-ROS1 translocation were detected in all three analyses by HC-NGS, providing evidence for the stability of the driver mutation. The other tumor sites (lymph nodes, lung, pleura) were in stable remission. The quality criteria for the clinically relevant regions were met [24,25]. The G2032R was not detected in the liver metastasis, which was the fastest progressing tumor site at first relapse (2nd NGS); however, it was detected in the plasma (3rd NGS) when the patient progressed on lorlatinib with the leading site again being the liver (Figure 2). Although it cannot be formally ruled out that the G2032R mutation may have stemmed from a different tumor site than the liver, it is highly improbable given the high allelic frequency of 11% at the second relapse. Since no supporting read of the G2032R was detected in the second NGS from the liver biopsy, the mutation most probably developed during therapeutic pressure of lorlatinib and became detecTable 6 weeks later at the time of liquid biopsy.

### 3.3. The Clinical Utility of Liquid Biopsy

For resistance monitoring the appropriate selection of an analysis method is crucial. NGS sequencing of tissue material provides high resolution molecular profiling; nevertheless, it might miss the representation of tumor heterogeneity or metastatic development [26,27]. In contrast, a liquid biopsy can represent tumor heterogeneity or detect multiple synchronous primaries; however, it strongly depends on the sample quality [28,29]. In this case report, by comparing the clinical utility of the second and the third NGS analyses, especially considering the non-invasiveness of blood-based testing, the advantages of a liquid biopsy are shown, as it clearly identified both the driver event and the resistance mutation.

### 3.4. TP53 as a Poor Prognosis Biomarker

The patient received crizotinib treatment in the first line of treatment and therapy was switched to lorlatinib after 4 months. Comparing the 4 months of our patient on crizotinib to the 22.4 months reported in the PROFILE001 study [30], she progressed dramatically fast. One of the reasons for fast progression may be the inactivating TP53 mutation, as such alterations might represent an intrinsic mode of TKI resistance and are suggested to have a negative predictive and prognostic role in ALK+ [12,18,31], as well as in EGFRmt+ NSCLC [13,14].

### 3.5. Therapeutic Options after G2032R

For patients that carry the ROS1 resistance mutation G2032R, therapeutic options are limited after lorlatinib therapy. Ceritinib, brigatinib, ensartinib and entrectinib have failed to demonstrate preclinical activity against the G2032R resistance mutation [2], and due to its toxicity, cabozantinib might not be preferred [32]. Lately, two promising novel drug candidates have emerged and might provide therapeutic options for ROS1+ patients with a G2032R mutation: repotrectinib (TPX-0005) and taletrectinib (DS-6051b, AB-106) showed potent preclinical activity [32,33,34] and are currently being evaluated in the TRIDENT-1 and TRUST-II clinical trials.

## 4. Conclusions

Here we report on a patient with a ROS1 translocation who developed a G2032R resistance mutation rarely observed under treatment on lorlatinib and detected by liquid biopsy. The patient’s clinical course was extraordinarily rapid highlighting that ROS1 translocations do not always purport a favorable prognosis, potentially due to concomitant TP53 mutation and the most prominent thrombotic diathesis. Further analyses should be undertaken to investigate the causes underlying this patient’s pathomechanism of disseminated intravasal coagulopathy. Whether TP53 mutations and thrombotic diathesis in and off themselves confer an inferior outcome should be prospectively elucidated.

## Data Availability

The data presented in this study are available on request from the corresponding author upon reasonable request.
